# Low-calorie sweeteners and health outcomes: an evaluation of rapid versus traditional evidence mapping

**DOI:** 10.1186/s13104-022-05926-3

**Published:** 2022-02-19

**Authors:** Juleen Lam, Rebecca Elmore, Brian Howard, Ruchir R. Shah

**Affiliations:** 1 Sciome LLC, 2 Davis Dr., Durham, NC 27709 USA; 2 California State University, East Bay, 25800 Carlos Bee Blvd., Hayward, CA 94542 USA

**Keywords:** Rapid evidence mapping, Evidence map, Systematic review, Evidence-based methodology, Low-calorie sweeteners, Artificial sweeteners

## Abstract

**Objective:**

Scientific evidence related to environmental exposures continues to mount. Tools such as evidence mapping support decision making, but can be resource- and time-intensive. We explored “rapid evidence mapping” to efficiently map scientific evidence using rigorous and transparent methodologies. We undertook a proof-of-concept case study on the topic of low-calorie sweeteners. Our intent was to conduct a traditional evidence map based on the same evidence base from a prior rapid evidence map case study to compare approaches, findings, and conclusions. We searched the literature, screened full text of studies, manually tagged and categorized articles, and created visualizations to map the evidence.

**Results:**

We conducted full-text screening of studies from the prior rapid evidence map and identified 255 relevant studies. Our findings corroborated those of the rapid evidence map, identifying most studies as short-term conducted in healthy individuals studying outcomes of appetite, energy sensing and body weight. We identified gaps in research areas related to outcomes of appetite and dietary intake, particularly in study populations with diabetes. Our findings illustrate the promise of rapid evidence mapping as a rigorous approach that can summarize scientific evidence, identify knowledge gaps, and identify areas for a future systematic review in a time-efficient manner.

**Supplementary Information:**

The online version contains supplementary material available at 10.1186/s13104-022-05926-3.

## Introduction

A recurring challenge in the field of environmental health is for stakeholders to efficiently summarize the ever-expanding scientific evidence and come to defendable scientific consensus to support decision-making [1–3]. Tools such as “evidence mapping” are frequently being used to identify the key areas of study along with important gaps in the literature [4]. These are gaining traction for its role informing risk management and policy decisions, and as a problem formulation tool to refine the focus of questions addressed in full systematic review [5]. However, evidence mapping can be a resource-intensive procedure, limiting its utility [6]. A more detailed overview of rEM and traditional evidence mapping is available elsewhere [4, 6–10].

We previously developed “rapid Evidence Mapping” (rEM), a knowledge synthesis approach where the evidence mapping review process is simplified in a resource-efficient manner [6]. We applied this to a case study on the topic of human dietary low-calorie sweeteners (LCS) exposures and select health outcomes [6]. However, in contrast to a more “traditional” evidence map approach, the rEM was based solely on title-and-abstract screening of references and relying on semi-automated machine learning to categorize and tag references.

Here, our main goal is to compare the rEM to traditional evidence mapping, by creating a traditional evidence map on the same evidence base used to generate the rEM and comparing the results as well as the time and resource commitments required for each.

## Main text

### Methods

To create the traditional evidence map, we started with the results from an rEM of human exposures to LCS published by Lam et al. [6] and applied the traditional evidence mapping approach (Additional file 1: Fig. S1). Our approach was outlined beforehand in our publicly available protocol [1].

#### Lam et al. [6] rEM approach

Lam et al. [6] searched PubMed using the keywords and MeSH terms provided in the Additional file 5, with date limit January 1, 1946–May 1, 2014. We utilized the same evidence base for direct comparison and did not update the search to identify newer studies. Lam et al. [6] defined the following inclusion/exclusion criteria (Additional file 2: Table S1): (1) randomized or non-randomized, controlled, clinical trials or prospective cohort study designs; (2) orally administered, FDA-approved or generally recognized as safe (GRAS) LCS; (3) at least one health outcome within five specified categories (Additional file 3: Table S2); (4) published in English; and (5) human subjects.

Lam et al. [6] imported all included titles and abstracts into SWIFT-Review (Sciome Workbench of Interactive computer-Facilitated Text-mining), a freely available text mining and machine learning software application (https://www.sciome.com/swift-review/). Software functionalities were used to automatically classify outcome, baseline health, and comparison categories [11] from reference abstracts, whereas manual review was conducted to extract study length and sample size categories.

#### Traditional evidence map approach

We started with all references included from Lam et al. [6] following title and abstract screening and conducted full text screening, applying the same inclusion/exclusion criteria. Screening was conducted in duplicate by two independent reviewers (JL, RE, LA, or DB) in SWIFT-Active Screener.

The following data were manually extracted from the full text of included studies:

- Study design characteristics (study design, study duration)
- Study population characteristics (baseline health status, age, sample size, anthropometrics)
- Study interventions/exposures and comparisons (type of LCS, comparison or control group)
- Number of people analyzed, form of administration
- Outcome information (all outcomes or endpoints of interest)
- Aim or hypothesis and funding source of the study

Data was extracted by a single reviewer (JL, RE, LA, or DB), with an independent second reviewer reviewing and verifying the data. A pilot test of 10 studies was conducted to calibrate screeners and resolve any confusion interpreting directions. Extracted data were limited to information available in the published manuscripts; additional details were not sought from study authors.

The same frequency tables and bubble plot visualizations generated by Lam et al. [6] were ultimately created based on the full-text extracted data (Additional file 1: Fig. S1).

### Results

**Fig. 1:**
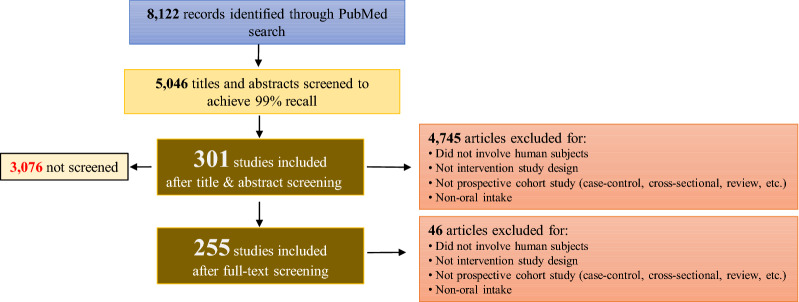
PRISMA (Preferred Reporting Items for Systematic Reviews and Meta-Analyses) diagram of study inclusion/exclusion process. This figure outlines the process by which 8122 unique records identified from a PubMed search implemented by Lam et al. (2019) were screened based on title and abstract for inclusion/exclusion up to 99% estimated recall. In this work, we screened the 301 studies included by Lam et al. (2019) based on full text and ultimately included 255 references

Lam et al.’s [6] original search retrieved 8122 unique records and ultimately included 301 references (Fig. 1). In our update, full-text screening of the 301 studies resulted in the ultimate inclusion of 255 references (Fig. 1). The following sections summarize the descriptive analyses of the data and compare the results of the rEM versus traditional evidence map.

#### Study characteristics and design

Lam et al. [6] reported that the majority of the 301 included studies were: (a) conducted in subjects where health status was healthy, mixed or other (83%); (b) interventions comparing LCS versus sugar (50%); and c) acute in duration lasting < 1 day (58%) (Table 1). Our conclusions based on the 255 full text screened and data extracted references were identical, although the proportion of studies falling in each category was slightly different—90%, 72%, and 31% respectively (Table 1).

**Table 1:** Summary of included studies (n = 255), and comparison to Lam et al. (2019) (n = 301)

	n (%)	
	This study	Lam et al. [6]
Length		
< 1 day	80 (31%)	174 (58%)
1–30 days	107 (42%)	53 (18%)
1–6 months	44 (17%)	41 (14%)
> 6 months	11 (4%)	19 (6%)
Unclear	13 (5%)	41 (14%)
Outcome groups*		
Energy sensing	152 (60%)	128 (43%)
Appetite	66 (26%)	70 (23%)
Glycemic	79 (31%)	61 (20%)
Dietary intake	68 (27%)	54 (18%)
Body weight/composition	23 (9%)	23 (8%)
Comparisons		
LCS vs. Sugar	183 (72%)	152 (50%)
LCS vs. Other	71 (28%)	133 (44%)
Unclear		16 (5%)
Baseline health*		
Healthy	192 (75%)	76 (25%)
Diabetes	11 (4%)	15 (5%)
Overweight	11 (4%)	12 (4%)
Mixed/Other	38 (15%)	174 (58%)
Unclear	3 (1%)	25 (8%)
Sample size**		
≤ 10	44 (17%)	31 (10%)
11–50	155 (61%)	118 (39%)
51–100	28 (11%)	21 (7%)
101–200	12 (5%)	12 (4%)
> 200	9 (4%)	10 (3%)
Unclear	7 (3%)	109 (36%)

*Studies can fall in multiple categories—therefore total % is greater than 100%

**Rounding errors may lead to total % greater than 100%

Among our 255 included studies, 60% reported energy sensing-related outcomes, 26% reported appetite-related outcomes, 31% reported glycemic-related outcomes, 27% reported dietary intake, and 9% reported body weight/composition-related outcomes (Table 1). In comparison, Lam et al. [6] reported these percentages as 43%, 23%, 20%, 18%, and 8% respectively (Table 1), consistently identifying smaller proportions in each category.

The majority of our included studies (61%) reported sample size ranging between 11 and 50 human participants. This aligned with findings from Lam et al. [6], although the proportion was smaller (39%). Few studies were reported to have larger sample sizes, for instance between 101 and 200 participants (we reported 5% and Lam et al. [6] 4%). One major difference was that Lam et al. [6] identified a large number of studies (36%) whose sample size was categorized as “unclear” whereas we only used this categorization for 3% of studies.

#### An evidence map to identify research gaps

We generated an evidence map consisting of frequency tables and bubble plots displaying the categorical features of included studies. Our results illustrate a consistent pattern with greater number of studies investigating comparisons of LCS versus sugars across all outcome categories. This difference was consistent, with the greatest difference in the body weight/composition outcome with 83% of studies comparing LCS vs. Sugars. The glycemic outcome demonstrated the lowest difference, with 70% of studies comparing LCS vs. Sugars. Lam et al. [6] reported a similar pattern, with the single exception of glycemic outcomes where the majority (56%) of studies categorized as LCS versus Others instead of LCS versus Sugars (44%).

We also categorized studies by study and outcome group (Additional file 4: Table S3). Our results illustrate that across four of the five outcome categories, the majority of studies were acute duration (either < 30 days). Very few studies were long-term (> 6 months). This was consistent with findings reported in Lam et al. [6].

**Fig. 2:**
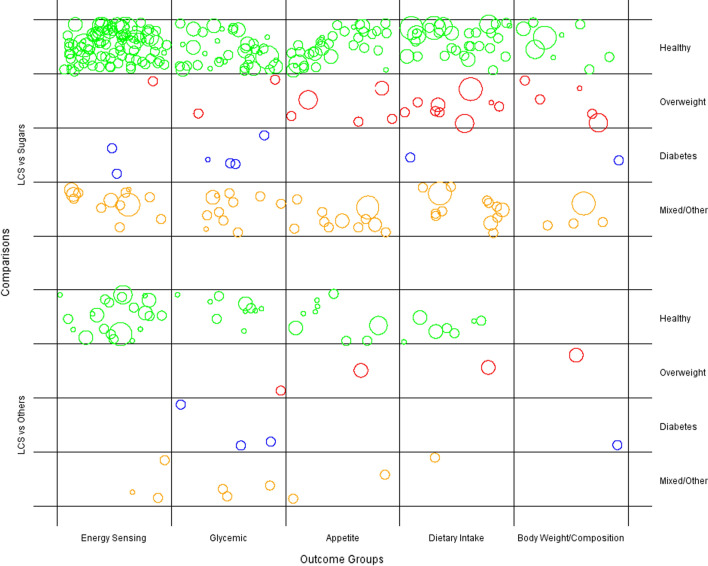
Bubble plot of included low-calorie sweetener studies by intervention type, health status, and outcome category. Data points are grouped and plotted according to study population baseline health status (healthy, overweight, diabetes, mixed/other) and outcome category, further stratified by intervention. Each data point represents a single study, and is randomly scattered in each grid to maximize visualization of the bubble; the size of each bubble indicates the sample size of the corresponding study, with larger bubbles representing larger study sample size. LCS vs. Sugars: An intervention comparison between low-calorie sweeteners and sugars; LCS vs. Others: An intervention comparison between low-calorie sweeteners and non-sugar sweetener. Mixed/Other: a mixture of healthy, overweight, diabetic, or unknown baseline health characteristic study population

The third component of the evidence map is a bubble plot (Fig. 2), where data points are grouped and plotted according to study population baseline health status and outcome category, stratified by intervention. Each data point represents a single study, and is randomly scattered in each grid (i.e., bubble position is meaningless). The size of each bubble indicates the sample size of the corresponding study, with larger bubbles representing larger study sample size (Lam et al. [6]).

Figure 2 illustrates that the majority of included studies utilize either generally healthy or mixed/other study populations compared to overweight and diabetic population types across all outcome categories. Most studies with diabetic participants report outcomes of either glycemic or body weight/composition. In contrast, there is a general lack of studies assessing appetite outcomes in people with diabetes, studies using LCS versus Sugars interventions to assess energy sensing and glycemic outcomes in overweight people, and studies comparing interventions LCS versus Others to assess energy sensing outcomes in both overweight and diabetic participants. These findings mirrored those reported by Lam et al. [6].

### Discussion

This case study was intended to explore the question of whether a rapid evidence map would produce similar conclusions based on a more traditional evidence map, while simultaneously providing an estimate of differences in time and resources required. To our knowledge, this is the first formal evaluation of rEM approaches compared to traditional evidence mapping. Our results indicate that rEM could be of great utility, particularly in the context of designing or implementing a systematic review. As evidence mapping gains visibility fields for their role in informing decision-making and risk management, our results outline promise for rEM approaches to play a significant role in increasing the efficiency of the policy-making process.

Our traditional evidence map initiated with the full text screening of references. An additional 46 studies (15%) were excluded at this step. This was not surprising—reviewing the full text provides additional details for reviewers to determine relevance. This illustrates one limitation of the rEM approach that an evidence map generated based on solely titles and abstracts of records will almost certainly include irrelevant references.

Our overall conclusions from the traditional evidence map corroborated those reported by Lam et al. [6]. Although there were minor discrepancies comparing our results with Lam et al. [6], the conclusions regarding the overarching question of this work as to where current research exists and where it is lacking was remarkably consistent. In general, most discrepancies were likely due to the greater precision from manually extracting data from the full text versus only the title and abstract. Furthermore, the utilization of the SWIFT-Review automated functionality to summarize and visualize data from the included abstracts by Lam et al. [6] further exacerbated the imprecision of data extracted from studies, combining issues with incomplete information included in the study abstracts versus full text along with the limitations of the automated software as compared to manual review and data extraction.

We estimated that the traditional evidence map required an additional 65 person-hours over the rEM approach. Although this additional required time may seem minimal, taken in the context of the relative size of the evidence base (i.e., n = 255) indicates that for a larger evidence base the potential time savings from implementing an rEM over a traditional evidence map could be significant.

### Conclusions

Alternative approaches to evidence mapping such as rEM are a promising approach to identify, collect, and evaluate scientific evidence that reduces the required time, effort, and resources. From our direct comparison to a traditional evidence map, we identified strong concordance with the ultimate conclusions when utilizing rEM. Since the main goal for evidence mapping is to quickly identify research gaps as well as opportunities for systematic review, an rEM may be able to do so in a more efficient manner that requires less time and resources.

### Limitations

Our traditional evidence map has several limitations, most of which could be readily addressed with increased time and resources. First, we only utilized references retrieved from searching one database (PubMed). By not including additional databases or the grey literature, we risk missing relevant studies. A broader literature search could have potentially retrieved a higher number of relevant studies and altered our final evidence map and conclusions.

We also utilized single-screening for records beyond the first 500 records (i.e., only one screener reviewing each reference as opposed to utilizing two independent reviewers). This reduced the time and resources required for screening, but introduces the possibility that a single screener could have erroneously excluded a relevant study. This limitation could be addressed by employing duplicate screening of references; however, this would have doubled the required time and resources.

Lastly, evidence mapping does not include the evaluation of study quality or risk of bias (i.e., internal validity). Thus, it is unknown whether the included studies are of high or poor quality. For instance, in categories where there was a high volume of included studies, it is possible that these studies are of variable quality. This also hinders limits our ability to state conclusions regarding the health effects of low calorie sweeteners; as expected, with an evidence map the end result is a visual depiction of where evidence exists and where it is lacking, not what conclusions one might ultimately draw regarding the body of scientific evidence. This could potentially be addressed by evaluating each included study using one of several available validated quality assessment tool; however, this would again require additional time and resources to complete this step.

## Supplementary Information

**Additional file 1.** A comparison between a traditional evidence map (Lam et al. 2022) and a rapid evidence map (Lam et al. 2019).

**Additional file 2.** List of inclusion and exclusion criteria for article screening.

**Additional file 3.** Outcome group categories defined by Lam et al. (2019) for included health outcomes.

**Additional file 4.** Table categorizing studies by study duration and outcome group comparing Lam et al. (2022) and Lam et al. (2019).

**Additional file 5.** Lam et al. (2019) search terms implemented in PubMed on January 24, 2018.

## Data Availability

The datasets used and/or analyzed during the current study are freely and publicly available from the corresponding author by request.

## Abbreviations

GRAS: Generally recognized as safe
LCS: Low-calorie sweeteners
PECO: Participants, exposure, comparator, and outcomes
rEM: Rapid evidence mapping
SWIFT: Sciome Workbench of Interactive computer-Facilitated Text-mining

## References

[1] EFSA (European Food Safety Authority). Application of systematic review methodology to food and feed safety assessments to support decision making. EFSA J. 2010. 10.2903/j.efsa.2010.1637 PMC13085193 42006960

[2] Rooney AA, Boyles AL, Wolfe MS, Bucher JR, Thayer KA (2014). Systematic review and evidence integration for literature-based environmental health science assessments. Environ Health Perspect.

[3] Woodruff TJ, Sutton P, Naviation Guide Working Group (2011). An evidence-based medicine methodology to bridge the gap between clinical and environmental health sciences. Health Aff.

[4] Miake-Lye IM, Hempel S, Shanman R, Shekelle PG (2016). What is an evidence map? A systematic review of published evidence maps and their definitions, methods, and products. Syst Rev.

[5] Wolffe TAM, Whaley P, Halsall C, Rooney AA, Walker VR (2019). Systematic evidence maps as a novel tool to support evidence-based decision-making in chemicals policy and risk management. Environ Int.

[6] Lam J, Howard BE, Thayer K, Shah RR (2019). Low-calorie sweeteners and health outcomes: a demonstration of rapid evidence mapping (rEM). Environ Int.

[7] Bragge P, Clavisi O, Turner T, Tavender E, Collie A, Gruen RL (2011). The global evidence mapping initiative: scoping research in broad topic areas. BMC Med Res Methodol.

[8] Grant MJ, Booth A (2009). A typology of reviews: an analysis of 14 review types and associated methodologies. Health Info Libr J.

[9] Khangura S, Konnyu K, Cushman R, Grimshaw J, Moher D (2012). Evidence summaries: the evolution of a rapid review approach. Syst Rev.

[10] Ganann R, Ciliska D, Thomas H (2010). Expediting systematic reviews: methods and implications of rapid reviews. Implement Sci.

[11] Howard BE, Phillips J, Miller K, Tandon A, Mav D, Shah MR (2016). SWIFT-review: a text-mining workbench for systematic review. Syst Rev.

