# Supplementary figures and images for: Low-calorie sweeteners and health outcomes: an evaluation of rapid versus traditional evidence mapping
Source: BMC Res Notes. 2022 Feb 19;15:65. doi: 10.1186/s13104-022-05926-3 (PMC8858516; doi:10.1186/s13104-022-05926-3)

Figure S1

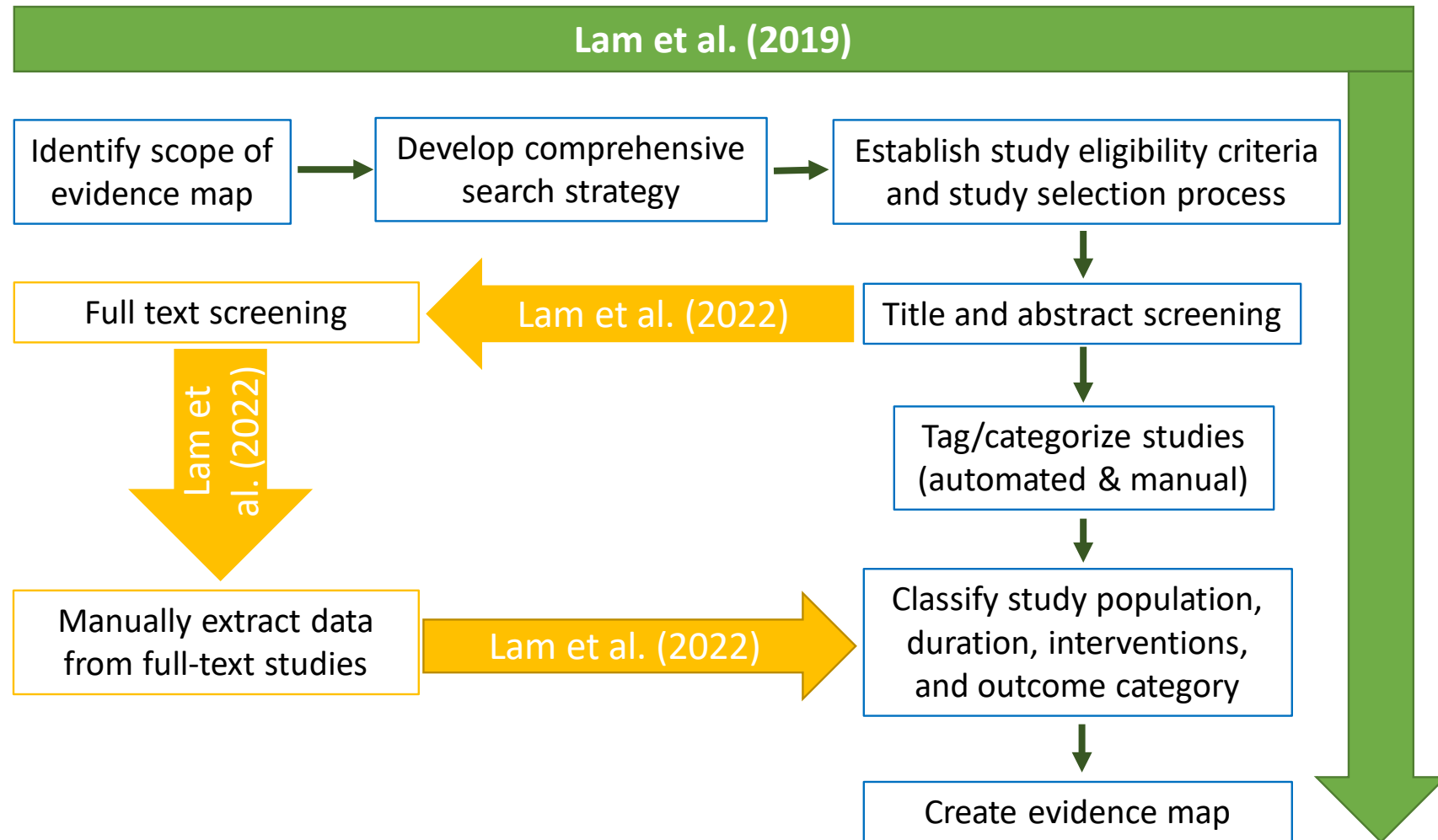

Supplement: Supplementary file 1 — Additional file 1. A comparison between a traditional evidence map (Lam et al. 2022) and a rapid evidence map (Lam et al. 2019). [file 13104_2022_5926_MOESM1_ESM.pdf]
