# Supplementary material for: Low-calorie sweeteners and health outcomes: an evaluation of rapid versus traditional evidence mapping
Source: BMC Res Notes. 2022 Feb 19;15:65. doi: 10.1186/s13104-022-05926-3 (PMC8858516; doi:10.1186/s13104-022-05926-3)
Supplement: Supplementary file 2 — Additional file 2. List of inclusion and exclusion criteria for article screening. [file 13104_2022_5926_MOESM2_ESM.pdf]

# Table S1

| Inclusion criteria | Exclusion Criteria |
|---|---|
| English language | Animal studies |
| Human subjects | In vitro cell studies |
| Intervention studies (randomized or non-randomized control trials) | Case control, cross-sectional studies, reviews, interview, bibliographies, letters, or guidelines |
| Prospective cohort studies | Systematic reviews and meta-analyses |
| Adults, pregnant women or infants (>6 months) | Cancer patients |
| FDA-approved or Generally Regarded as Safe sweeteners | Non-oral intake |
