# Supplementary material for: Low-calorie sweeteners and health outcomes: an evaluation of rapid versus traditional evidence mapping
Source: BMC Res Notes. 2022 Feb 19;15:65. doi: 10.1186/s13104-022-05926-3 (PMC8858516; doi:10.1186/s13104-022-05926-3)
Supplement: Supplementary file 3 — Additional file 3. Outcome group categories defined by Lam et al. (2019) for included health outcomes. [file 13104_2022_5926_MOESM3_ESM.pdf]

Table S2

Outcome Groups	Included Outcomes
Appetite	Appetite ratings using a visual analog scale (VAS), hunger, desire to eat, fullness, prospective consumption, thirst, motivational and behavioral factors reported through questionnaire
Energy sensing by brain	Neurological measurements (fMRI, EEG), sensory ratings (sweetness, intensity, pleasantness, sensory specific satiation), taste, perception and preference, taste reaction time
Body weight or body composition	Body weight, body composition, BMI, waist circumferences, weight, or BMI changes
Dietary intake	Energy intake, dietary intake, food intake, carbohydrate intake, sugar intake, salt intake, water intake
Glycemic	Glucose, Hemoglobin A1c (HbA1c), insulin concentration, insulin sensitivity, hypoglycemia, glucagon, glucose-dependent insulinotropic peptide (GIP), glucagon-like peptide-1 (GLP-1), peptide tyrosine tyrosine (PYY), cholecystokinin (CCK), enterostatin, ghrelin, leptin, somatostatin, oxyntomodulin
