# Supplementary material for: Low-calorie sweeteners and health outcomes: an evaluation of rapid versus traditional evidence mapping
Source: BMC Res Notes. 2022 Feb 19;15:65. doi: 10.1186/s13104-022-05926-3 (PMC8858516; doi:10.1186/s13104-022-05926-3)
Supplement: Supplementary file 4 — Additional file 4. Table categorizing studies by study duration and outcome group comparing Lam et al. (2022) and Lam et al. (2019). [file 13104_2022_5926_MOESM4_ESM.pdf]

Table S3

	Energy Sensing		Glycemic*		Appetite		Dietary Intake*		Body Weight/ Composition	
	Lam et al. (2022)	*Lam et al. (2019)*	*Lam et al. (2022)*	*Lam et al. (2019)*	*Lam et al. (2022)*	*Lam et al. (2019)*	*Lam et al. (2022)*	*Lam et al. (2019)*	*Lam et al. (2022)*	*Lam et al. (2019)*
**> 6 Months**	<1% (n=1)	1% (n=1)	4% (n=3)	2% (n=1)	3% (n=2)	1% (n=1)	7% (n=5)	2% (n=1)	26% (n=6)	26% (n=6)
**1-6 Months**	11% (n=17)	1% (n=1)	19% (n=15)	10% (n=6)	24% (n=16)	9% (n=6)	37% (n=25)	6% (n=3)	52% (n=12)	30% (n=7)
**1-30 Days**	37% (n=56)	12% (n=15)	65% (n=51)	30% (n=18)	56% (n=37)	24% (n=17)	44% (n=30)	33% (n=18)	22% (n=5)	35% (n=8)
**< 1 Day**	46% (n=70)	72% (n=92)	9% (n=7)	57% (n=35)	14% (n=9)	60% (n=42)	7% (n=5)	59% (n=32)	0% (n=0)	4% (n=1)
**Unclear/ not stated**	5% (n=8)	15% (n=19)	4% (n=3)	2% (n=1)	3% (n=2)	6% (n=4)	4% (n=3)	0% (n=0)	0% (n=0)	4% (n=1)
**TOTAL # STUDIES**	**152**	**128**	**79**	**61**	**66**	**70**	**68**	**54**	**23**	**23**

* Rounding may lead to total % greater than 100%
