# Supplementary material for: Low-calorie sweeteners and health outcomes: an evaluation of rapid versus traditional evidence mapping
Source: BMC Res Notes. 2022 Feb 19;15:65. doi: 10.1186/s13104-022-05926-3 (PMC8858516; doi:10.1186/s13104-022-05926-3)
Supplement: Supplementary file 5 — Additional file 5. Lam et al. (2019) search terms implemented in PubMed on January 24, 2018. [file 13104_2022_5926_MOESM5_ESM.pdf]

Supplemental file 1

Lam et al. (2019) search terms implemented
in PubMed on January 24, 2018

((((((((((((("acesulfame K" or "low calorie sweetener" or "artificial sweetener" or Sweetening Agents [mh] or "acesulfame potassium" or advantame or alitame or aspartame or Aspartame[mh] or cyclamate or Cyclamates[mh] or "D-tagatose" or erythritol or Erythritol[mh] or "high intensity sweetener" or Non-Nutritive Sweeteners[mh] or "high potency sweetener" or "hydrogenated starch hydrolysate" or "maltitol syrup" or "sorbitol syrup" or Sorbitol[mh] or "intense sweetener" or isomaltulose or isomalt or lactitol or "Luo han guo extract" or "cucurbitane glycosides" or mogroside or maltitol or mannitol or Mannitol[mh] or neotame or "nonnutritive sweeteners" or polyol or "rebaudioside A" or rebiana or saccharin or Saccharin[mh] or sorbitol or Sorbitol[mh] or stevioside or "stevia extract" or "stevia leaf extract" or sucralose or "trichlorogalactosucrose" or "sugar alcohol" or Sugar Alcohols[mh] or trehalose or Trehalose[mh] or xylitol or Xylitol[mh] or "sweetening agent" or Sweetening Agents[mh] or "sugar substitute" or Sweetening Agents[mh] or "diet beverage" or "diet drink" or "diet soda" or "diet soft drink" or "low calorie sweetener" or "no sugar added" or "non-caloric beverage" or "non caloric drink" or "non caloric soft drink" or "reduced sugar" or "sugar free" or "Reduced calorie beverage" or "reduced sugar beverage" or "artificially sweetened")) AND ("cephalic phase responses" or Brain[mh] or "immunoglobulins" or Immunoglobulins[mh] or "dorsal motor nucleus of the vagus" or "nucleus of the solitary tract" or Solitary Nucleus[mh] or "lateral hypothalamus" or Ventromedial Hypothalamic Nucleus[mh] or "ventromedial hypothalamus" or Orexin Receptors[mh] or orexins or "thyrotropin-releasing hormone" or Thyrotropin-Releasing Hormone[mh] or Amygdala[mh] or "Amygdala response" or "Blood oxygen level-dependent" or Taste[mh] or "Taste" or "Flavor" or "visual analog score" or "Oral somatosensation" or "Retronasal olfaction" or "Taste perception" or Taste Perception[mh] or "taste physiology" or "Choice behavior" or Choice Behavior[mh] or "Food habits" or Food Habits[mh] or "Food preferences" or Food Preferences[mh] or "Instinct" or Instinct[mh] or "Flavor processing" or "Nucleus accumbens" or Nucleus Accumbens[mh] or "opioid" or Analgesics, Opioid[mh] or "ventral pallidum" or limbic or mesolimbic or Dopamine[mh] or dopamine or Leptin[mh] or leptin or "brain mapping" or Brain Mapping[mh] or "magnetic resonance imaging" or Magnetic Resonance Imaging[mh]or "positron-emission tomography" or Positron-Emission Tomography[mh])) AND ("0001/01/01"[PDat] : "2014/05/01"[PDat]) AND Humans[Mesh] AND English[lang])) NOT (case control study[mh] or "case control" or Cross-Sectional Studies[mh] or "cross-sectional" or "prevalence study")) AND ("0001/01/01"[PDat] : "2014/05/01"[PDat]) AND Humans[Mesh] AND English[lang])) OR (((((((("acesulfame K" or "low calorie sweetener" or "artificial sweetener" or Sweetening Agents [mh] or "acesulfame potassium" or advantame or alitame or aspartame or Aspartame[mh] or cyclamate or Cyclamates[mh] or "D-tagatose" or erythritol or Erythritol[mh] or "high intensity sweetener" or Non-Nutritive Sweeteners[mh] or "high potency sweetener" or "hydrogenated starch hydrolysate" or "maltitol syrup" or "sorbitol syrup" or Sorbitol[mh] or "intense sweetener" or isomaltulose or isomalt or lactitol or "Luo han guo extract" or "cucurbitane glycosides" or mogroside or maltitol or mannitol or Mannitol[mh] or neotame or "nonnutritive sweeteners" or polyol or "rebaudioside A" or rebiana or saccharin or Saccharin[mh] or sorbitol or Sorbitol[mh] or stevioside or "stevia extract" or "stevia leaf extract" or sucralose or "trichlorogalactosucrose" or "sugar alcohol" or Sugar Alcohols[mh] or trehalose or Trehalose[mh] or xylitol or Xylitol[mh] or "sweetening agent" or Sweetening Agents[mh] or "sugar substitute" or Sweetening Agents[mh] or "diet beverage" or "diet drink" or "diet soda" or "diet soft drink" or "low calorie sweetener" or "no sugar added" or "non-caloric beverage" or "non caloric drink" or "non caloric soft drink" or "reduced sugar" or "sugar free" or "Reduced calorie beverage" or "reduced sugar beverage" or "artificially sweetened") AND ("0001/01/01"[PDat] : "2014/05/01"[PDat]) AND Humans[Mesh] AND English[lang])) AND ("Eating disorders" or Eating Disorders[mh] or "Analgesics, opioid" or Analgesics, Opioid[mh] or "Brain-derived neurotrophic factor" or Brain-Derived Neurotrophic Factor[mh] or "Cannabinoid receptor modulators" or Cannabinoid Receptor Modulators[mh] or Ghrelin[mh] or "Ghrelin" or "Reward" or Reward[mh] or "Impulsivity" or Obsessive-Compulsive Disorder[mh] or "Obsessive-compulsive" or Bulimia[mh] or "Bulimic" or Impulsive Behavior[mh] or "impulsive")) AND ("0001/01/01"[PDat] : "2014/05/01"[PDat]) AND Humans[Mesh] AND English[lang])) NOT (case control study[mh] or "case control" or Cross-Sectional Studies[mh] or "cross-sectional" or "prevalence study")) AND ("0001/01/01"[PDat] : "2014/05/01"[PDat]) AND Humans[Mesh] AND English[lang])) OR ((((("acesulfame K" or "low calorie sweetener" or "artificial sweetener" or Sweetening Agents [mh] or "acesulfame potassium" or advantame or alitame

or aspartame or Aspartame[mh] or cyclamate or Cyclamates[mh] or "D-tagatose" or erythritol or Erythritol[mh] or "high intensity sweetener" or Non-Nutritive Sweeteners[mh] or "high potency sweetener" or "hydrogenated starch hydrolysate" or "maltitol syrup" or "sorbitol syrup" or Sorbitol[mh] or "intense sweetener" or isomaltulose or isomalt or lactitol or "Luo han guo extract" or "cucurbitane glycosides" or mogroside or maltitol or mannitol or Mannitol[mh] or neotame or "nonnutritive sweeteners" or polyol or "rebaudioside A" or rebiana or saccharin or Saccharin[mh] or sorbitol or Sorbitol[mh] or stevioside or "stevia extract" or "stevia leaf extract" or sucralose or "trichlorogalactosucrose" or "sugar alcohol" or Sugar Alcohols[mh] or trehalose or Trehalose[mh] or xylitol or Xylitol[mh] or "sweetening agent" or Sweetening Agents[mh] or "sugar substitute" or Sweetening Agents[mh] or "diet beverage" or "diet drink" or "diet soda" or "diet soft drink" or "low calorie sweetener" or "no sugar added" or "non-caloric beverage" or "non caloric drink" or "non caloric soft drink" or "reduced sugar" or "sugar free" or "Reduced calorie beverage" or "reduced sugar beverage" or "artificially sweetened")) AND ("Gustatory pathway" or "energy homeostasis" or "fMRI" or Magnetic Resonance Imaging[mh] or "PET" or "Functional brain imaging" or Functional Neuroimaging[mh] or "Activation likelihood estimation" or Incretins[mh] or Gastric Inhibitory Polypeptide[mh] or Glucagon-Like Peptide 1[mh] or "Incretin hormones" or "peptide YY" or Peptide YY[mh] or "cholecystokinin" or Cholecystokinin[mh] or "pancreatic polypeptide" or Pancreatic Polypeptide[mh] or "amylin" or "oxyntomodulin" or Oxyntomodulin[mh])) NOT (case control study[mh] or "case control" or Cross-Sectional Studies[mh] or "cross-sectional" or "prevalence study")) AND ("0001/01/01"[PDat] : "2014/05/01"[PDat]) AND Humans[Mesh] AND English[lang])) OR ((((("acesulfame K" or "low calorie sweetener" or "artificial sweetener" or Sweetening Agents [mh] or "acesulfame potassium" or advantame or alitame or aspartame or Aspartame[mh] or cyclamate or Cyclamates[mh] or "D-tagatose" or erythritol or Erythritol[mh] or "high intensity sweetener" or Non-Nutritive Sweeteners[mh] or "high potency sweetener" or "hydrogenated starch hydrolysate" or "maltitol syrup" or "sorbitol syrup" or Sorbitol[mh] or "intense sweetener" or isomaltulose or isomalt or lactitol or "Luo han guo extract" or "cucurbitane glycosides" or mogroside or maltitol or mannitol or Mannitol[mh] or neotame or "nonnutritive sweeteners" or polyol or "rebaudioside A" or rebiana or saccharin or Saccharin[mh] or sorbitol or Sorbitol[mh] or stevioside or "stevia extract" or "stevia leaf extract" or sucralose or "trichlorogalactosucrose" or "sugar alcohol" or Sugar Alcohols[mh] or trehalose or Trehalose[mh] or xylitol or Xylitol[mh] or "sweetening agent" or Sweetening Agents[mh] or "sugar substitute" or Sweetening Agents[mh] or "diet beverage" or "diet drink" or "diet soda" or "diet soft drink" or "low calorie sweetener" or "no sugar added" or "non-caloric beverage" or "non caloric drink" or "non caloric soft drink" or "reduced sugar" or "sugar free" or "Reduced calorie beverage" or "reduced sugar beverage" or "artificially sweetened")) AND (Satiation[mh] or Satiety Response[mh] or Appetite Regulation[mh] or "addictive behavior" or addictive behavior[mh] or "uncontrollable eating" or Obesity, Morbid[mh] or Eating Disorders[mh] or Bulimia[mh] or "abusive eating" or Eating Disorders[mh] or "excessive craving" or Behavior, Addictive[mh] or "binge eating" or Bulimia[mh] or Energy Intake[mh] or Ghrelin[mh] or Leptin[mh] or "total peptide tyrosine" or "total peptide tyrosine-tyrosine" or "tyrosine" or "PYY" or "total glucagon-like peptide-1" or "GLP-1" or "GLP1" or "glucagon like peptide 1")) NOT (case control study[mh] or "case control" or Cross-Sectional Studies[mh] or "cross-sectional" or "prevalence study")) AND ("0001/01/01"[PDat] : "2014/05/01"[PDat]) AND Humans[Mesh] AND English[lang])) OR ((((("acesulfame K" or "low calorie sweetener" or "artificial sweetener" or Sweetening Agents [mh] or "acesulfame potassium" or advantame or alitame or aspartame or Aspartame[mh] or cyclamate or Cyclamates[mh] or "D-tagatose" or erythritol or Erythritol[mh] or "high intensity sweetener" or Non-Nutritive Sweeteners[mh] or "high potency sweetener" or "hydrogenated starch hydrolysate" or "maltitol syrup" or "sorbitol syrup" or Sorbitol[mh] or "intense sweetener" or isomaltulose or isomalt or lactitol or "Luo han guo extract" or "cucurbitane glycosides" or mogroside or maltitol or mannitol or Mannitol[mh] or neotame or "nonnutritive sweeteners" or polyol or "rebaudioside A" or rebiana or saccharin or Saccharin[mh] or sorbitol or Sorbitol[mh] or stevioside or "stevia extract" or "stevia leaf extract" or sucralose or "trichlorogalactosucrose" or "sugar alcohol" or Sugar Alcohols[mh] or trehalose or Trehalose[mh] or xylitol or Xylitol[mh] or "sweetening agent" or Sweetening Agents[mh] or "sugar substitute" or Sweetening Agents[mh] or "diet beverage" or "diet drink" or "diet soda" or "diet soft drink" or "low calorie sweetener" or "no sugar added" or "non-caloric beverage" or "non caloric drink" or "non caloric soft drink" or "reduced sugar" or "sugar free" or "Reduced calorie beverage" or "reduced sugar beverage" or "artificially sweetened")) AND (Weight Loss[mh] or Body Weight Changes[mh] or Body Weight[mh] or Weight Gain[mh] or Obesity[mh] or Overweight[mh] or Obesity Hypoventilation Syndrome[mh] or Obesity, Abdominal[mh] or Body Constitution or "body weights and measures" or Body Fat Distribution[mh] or Adiposity[mh] or Body Mass Index[mh] or Body Size[mh] or Waist

Circumference[mh] or Waist-Hip Ratio[mh] or Skinfold Thickness[mh])) NOT (case control study[mh] or "case control" or Cross-Sectional Studies[mh] or "cross-sectional" or "prevalence study")) AND ("0001/01/01"[PDat] : "2014/05/01"[PDat]) AND Humans[Mesh] AND English[lang]))
